# Epidemiology of extrapulmonary tuberculosis in Brazil: a hierarchical model

**DOI:** 10.1186/1471-2334-14-9

**Published:** 2014-01-08

**Authors:** Teresa Gomes, Bárbara Reis-Santos, Adelmo Bertolde, John L Johnson, Lee W Riley, Ethel Leonor Maciel

**Affiliations:** 1 Laboratory of Epidemiology, Universidade Federal do Espírito Santo, Vitória, ES, Brazil; 2 Departament of Statistics, Universidade Federal do Espírito Santo, Vitória, ES, Brazil; 3 Tuberculosis Research Unit, Department of Medicine, Division of Infectious Diseases, Case Western Reserve University, Cleveland, OH, USA; 4 Division of Infectious Disease and Vaccinology, School of Public Health, University of California, Berkeley, CA, USA

## Abstract

**Background:**

Although extrapulmonary tuberculosis (EPTB) is less frequent than Pulmonary Tuberculosis (PTB) and is a secondary target for national TB control programs, its significance has increased worldwide during the HIV epidemic. The objective of this study was to examine the epidemiology of EPTB in Brazil between 2007 and 2011.

**Methods:**

Cross-sectional study involving all cases of TB reported to the Brazilian Notifiable Diseases Surveillance System (Sistema de Informações de Agravo de Notificação - SINAN) in Brazil between 2007 and 2011. Sociodemographic and clinical characteristics of patients with exclusively PTB and exclusively EPTB were compared. Following analysis with Pearson’s chi-square test, variables with p < 0.05 were included in a hierarchical regression model. Variables with p < 0.05 in the corresponding level were kept in the model.

**Results:**

A total of 427,548 cases of TB were included. Of these, 356,342 cases (83.35%; 95% confidence interval (CI) 83.23% - 83.45%) were PTB, 57,217 (13.37%; 95% CI 13.28% - 13.48%) were EPTB, 13,989 (3.27%; 95% CI 3.21% - 3.32%) were concurrent pulmonary and extrapulmonary TB. Patients with EPTB were mainly white (16.7%), and most (29.1%) patients had five to eight years of education. Among comorbidities, HIV infection was prominent (OR 2.15; 95% CI 2.09 – 2.21), although the proportion of cases awaiting test results or untested was high (39%). Ethanol use (OR 0.45; 95% CI 0.43 – 0.46), diabetes mellitus (OR 0.54; 95% CI 0.51 – 0.57) and mental illness (OR 0.88; 95% CI 0.82 – 0.95) were associated with PTB.

**Conclusions:**

Thirteen percent of patients diagnosed with TB in Brazil have only EPTB. More effective diagnostic strategies and control measures are needed to reduce the number of cases of extrapulmonary TB in Brazil.

## Background

Although data are limited, extrapulmonary disease is a common presentation of tuberculosis (TB) in Brazil. In 2011, 14% of all cases of TB reported to the World Health Organization (WHO) were extra-pulmonary TB (EPTB) [1].

A national literature search demonstrated that the epidemiology of EPTB is infrequently mentioned. EPTB is rarely included in publications on the epidemiological profile of TB [2,3]. Two studies at the national level mention EPTB only when discussing TB in persons co-infected with human immunodeficiency virus (HIV) [4,5]. Studies have addressed EPTB at the level of states [6], municipalities [4,7], and healthcare services [5,8] in Brazil, but none have addressed it at the national level. This situation is illustrated by the omission of EPTB from the latest epidemiological report on TB by the Brazilian Health Ministry published in 2012 [9].

The rate of EPTB in Brazil increased from 6.8 per 100,000 people in 1981 to 7.0 per 100,000 people in 1991. A study conducted in the municipality of Campinas, São Paulo between 2001 and 2009 found a 23.7% reduction in the number of cases of pulmonary TB (PTB) but only a 5.9% reduction in the number of cases of EPTB [4].

One of the main factors affecting the rate of EPTB in Brazil is the HIV/AIDS epidemic because the development of EPTB is favored by HIV infection and other conditions that suppress immune function [10].

Due to the impact of the HIV epidemic on EPTB, the scarcity of national studies of EPTB, and the relevance of EPTB to the epidemiology of TB and TB control in Brazil, the present study was done to examine the epidemiology of EPTB in Brazil during the five year period from 2007 to 2011.

## Methods

Information systems are essential tools for the assessment and planning of health actions. Cases of TB are recorded by the Brazilian Notifiable Diseases Surveillance System [Sistema de Informação de Agravos de Notificação (SINAN)] based on data abstracted from forms used in the investigation and follow-up of cases. SINAN is the main survey system for collection and analysis of national data on TB in Brazil [11,12].

A cross-sectional study was performed including all cases of TB in Brazil reported to SINAN from January 1, 2007 to December 31, 2011. Lack of data in the variable “clinical form of TB” on SINAN was the only reason for exclusion from the study.

Information about the clinical form of TB is a required field on SINAN survey system. This information is entered by a healthcare provider at the time of notification of a case of TB when the diagnosis of TB has been confirmed. Although all Brazilian municipalities transmit data to SINAN, only 70% enter their data electronically. Database update at higher hierarchical levels is routinely conducted through vertical data transfers from the municipality to the state and then onwards to the Federal level [13]. Because TB diagnosis and treatment is free of charge in Brazil and because patients must be notified in SINAN before anti-TB treatment is started, reporting of TB cases to SINAN and the national TB program is reasonably complete in Brazil.

Clinical forms of TB were classified using WHO definitions [14]. PTB was defined as TB affecting the lung parenchyma. EPTB was defined as TB affecting organs and tissues outside of the lungs (e.g. skin, pleura, bones and joints, meninges, etc.). Miliary TB was classified as extrapulmonary TB. Information on sites of extrapulmonary disease (pleural, lymphatic, genitourinary, bone, eye, skin, laryngeal, miliary, meningitis and others) is routinely captured in the SINAN data base [15].

First, a descriptive analysis of the absolute and relative frequencies of patients presenting with pulmonary or extrapulmonary TB, both pulmonary and extrapulmonary TB, and disease at various extrapulmonary sites (pleural, lymphatic, genitourinary, bone, eye, skin, miliary and others) was done. Then, characteristics of cases of exclusively EPTB and exclusively PTB were compared. Cases of PTB + EPTB were excluded from this stage because these forms of TB affect pulmonary and extrapulmonary sites simultaneously, and the cases could not be allocated to either group leading to possible classification bias. Excluding such cases is similar to methods used in a recent national study of extrapulmonary TB in the USA [16].

Pearson’s chi-square test was used to compare the rates of PTB and EPTB. Variables with a P value < 0.05 were included in a hierarchical logistic regression model.

Study variables were assessed and allocated into five levels: Level 1 (sociodemographic) variables included gender (female or male), age (0 to 14 years, 15 to 24 years, 25 to 34 years, 35 to 44 years, 45 to 54 years, 55 to 64 years, more than 65 years), ethnicity (white, black, brown, Asian or Indian), and educational level (illiterate, 1 to 4 years of education, 5 to 8 years, 9 to 12 years, greater than 12 years, non-applicable); Level 2 (environmental) variables included residence (urban, rural, or peri-urban), and institutionalization [yes (prison, nursing home, orphanage, hospital, psychiatric institution, or others) or no]. Level 3 (previous comorbidities) variables included HIV/AIDS (negative or positive), alcoholism (yes or no), diabetes (yes or no), mental illness (yes or no), and other diseases (yes or no). Level 4 (clinical features) variables included admission type (new case, relapse, readmission following dropout, unknown, or transfer), tuberculin skin test (TST) [positive (>5 mm) or negative (0 to 4 mm)], sputum smear test for acid-fast bacilli (AFB) (negative or positive), culture (positive or negative), histopathology [positive (positive AFB and suggestive of TB) or negative TB. Level 5 (treatment outcome of TB) variables included anti-TB treatment administered under Directly Observed Therapy (DOT) (yes or no) and outcome status [cure, dropout, death by TB, death due to other causes, transfer, or multi-drug resistant TB (MDR-TB)].

Tuberculosis is a disease with a complex causal chain. Constructing a hierarchical model may be a better way to capture the interrelationships between its determinants. In this model, variables are included from distal to proximal ones, according to different levels of a causal network arising from a robust theoretical base [17,18]. Associations resulting from the hierarchical regression model are adjusted for the variables in the same level and those in previous levels, taking into account both confounders and mediators [17,18].

The present model was based on the conceptual framework for social determinants of TB formulated by Maciel [19]. Independent variables were hierarchized into five levels: socio-demographic (Level 1), environmental (Level 2), previous comorbidities (Level 3), clinical features of TB (Level 4), and outcome of anti-TB treatment (Level 5), as outlined above.

Distinct models were used in building each level and the variables that exhibited an association with the clinical form of TB (p ≤ 0.05) at each level were included in the subsequent level, resulting in the following models: level 1 – sociodemographic characteristics; level 2 – variables included from level 1 + environmental characteristics; level 3 – variables included from level 2 + comorbidities; level 4 – variables included from level 3 + clinical characteristics of TB; and level 5 – variables included from level 4 + outcome variables.

Microsoft Excel 2010 software was used to construct the databases and generate the graphs. Statistical analysis was performed with Stata version 11.0, and included calculation of odds ratios (OR) with 95% confidence intervals (95% CI).

The study was approved by the research ethics committee of the Center of Health Science of the Federal University of Espírito Santo (UFES) - Record Number 121/06. The institutional review board of UFES granted permission for use of information from the SINAN database for the purposes of the study and waived the need for written informed consent from participants as the study involved only secondary data and the confidentiality of the patients’ identities was protected.

## Results

### Study population

**Figure 1:**
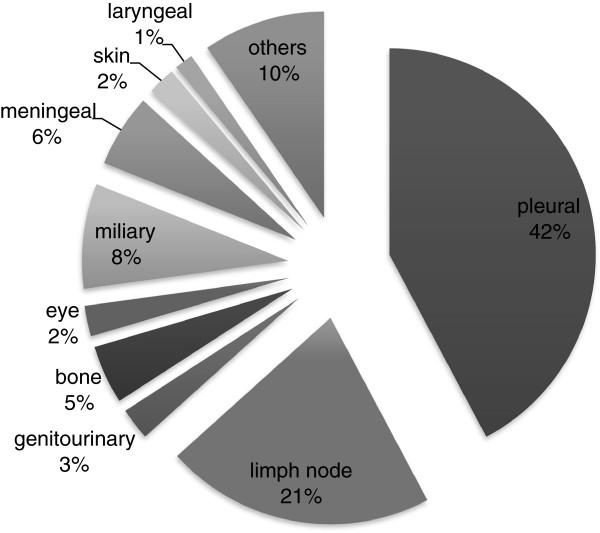
Clinical sites of extrapulmonary tuberculosis in Brazil, 2007 to 2011.

Between 2007 and 2011, 428,039 cases of TB were reported to SINAN. Of these, 491 (0.11%) were excluded because the clinical form of TB was not entered into SINAN, resulting in a final sample of 427,548 cases. The sample included 356,342 cases of PTB (83.35%; 95% CI 83.23% - 83.45%), 57,217 cases of EPTB (13.37%; 95% CI 13.28% - 13.48%), 13,989 cases of PTB + EPTB (3.27%; 95% CI 3.21% - 3.32%). The total number of cases of TB with some type of extrapulmonary disease (all forms except PTB) was 71,207. In 70,131 of such cases, the clinically affected sites were recorded in SINAN. Their relative proportions are depicted in Figure 1. Only the first site of disease reported is depicted in the figure. In 4,307 (6%) cases of EPTB or PTB + EPTB, a second site was also involved. Among these secondary’s sites of TB, 27.8% involved lymph nodes, 18.8% pleural, 10.5% miliary, 8.4% laryngeal, 5.4% meningeal, 4.4% bone, 3.3% skin, 2.6% genitourinary, 1.1% ocular and 17.7% others.

The following characteristics of patients with EPTB were prominent in the descriptive analysis: male gender (60.7%), age of 0 to 15 years old (24.6%), white race (16.7%), and history of five to eight years of education (29.1%). The proportion of cases without HIV result was 39.2%; furthermore, 50.2% of the patients were not treated under DOT and 22.4% had no information about this variable, 9.4% of the cases were transferred out, and 71.5% were cured (data not shown). Other features of the cases of EPTB compared to PTB are described in Tables 1 and 2.

**Table 1:** Sociodemographic characteristics of EPTB and PTB in Brazil, 2007 – 2011

Characteristics N*	**Categories**	**EPTB (%) (n = 53,853)**	**PTB (%) (n = 356,341)**	P value**
Gender	Female	22,472 (39.3)	115,541 (32.4)	<0.001
(413,533)	Male	34,743 (60.7)	240,777 (67.6)	
Age	0-14 years old	3,743 (6.4)	10,733 (3.0)	<0.001
(409,981)	15– 24 years old	7,543(14.0)	60,798 (17.1)	
	25– 34 years old	13,219 (24.6)	85,045(23.9)	
	35 – 44 years old	11,349 (21.2)	70,565 (19.8)	
	45 – 54 years old	8,616 (16.0)	61,198 (17.2)	
	55 – 64 years old	5,040 (9.4)	36,748 (10.3)	
	>65 years old	4,545 (8.4)	31,064 (8.7)	
Skin color/race	White	22,474 (45.2)	112,112 (36.0)	<0.001
(361,362)	Black	6,333 (12.8)	47,566 (15.3)	
	Brown	19,791 (39.9)	144,380 (46.3)	
	Asian	557 (1.1)	3,531 (1.1)	
	Indigenous	506 (1.0)	4,112 (1.3)	
Education	Illiterate	1,432 (4.1)	18,873 (8.4)	<0.001
(258,978)	1 – 4 years	8,662 (24.6)	77,014 (34.4)	
	5 – 8 years	10,242 (29.1)	69,786 (31.2)	
	9 – 12 years	8,493 (24.2)	39,089 (17.5)	
	>12 years	4,490 (12.8)	11,818 (5.3)	
	Non-applicable	1,818 (5.2)	7,261 (3.2)	
Dwelling	Urban	37,948 (91.3)	241,956 (88.2)	<0.001
(315,989)	Rural	3,311 (8.0)	30,260 (11.0)	
	Peri-urban	273 (0.7)	2,241 (0.8)	
Institutionalization	No	45,605 (93.3)	270,977 (88.8)	<0.001
(354,189)	Yes	3,249 (6.7)	34,358 (11.2)	

*Number of valid observations. **Pearson’s chi-squared test.

EPTB: Extrapulmonary tuberculosis. PTB: pulmonary tuberculosis.

**Table 2:** Comorbidities, clinical features, and treatment outcomes of EPTB and PTB in Brazil, 2007- 2011

Characteristics N*	**Categories**	**EPTB (%) (n = 53,853)**	**PTB (%) (n = 356,341)**	P value**
HIV/AIDS	Negative	24,072 (69.2)	149,526 (83.8)	<0.001
(213,336)	Positive	10,755 (30.8)	28,983 (16.2)	
Alcoholism	No	46,056(91.6)	249,995 (82.0)	<0.001
(355,292)	Yes	4,236 (8.4)	55,005 (18.0)	
Diabetes	No	48,121 (96.1)	279,415 (92.9)	<0.001
(350,756)	Yes	1,960 (3.9)	21,260 (7.1)	
Mental illness	No	49,114 (98.1)	293,039 (97.3)	<0.001
(351,236)	Yes	960 (1.9)	8,123 (2.7)	
Other diseases	No	38,142 (83.8)	229,706 (84.7)	0.001
(316,784)	Yes	7,357 (16.2)	41,579 (15.3)	
Admission type	New cases	50,126 (87.6)	290,431 (81.5)	<0.001
(413,559)	Relapse	2,178 (3.8)	23,013 (6.5)	
	Readmission after dropout	1,386(2.4)	23,191 (6.5)	
	Unknown	151 (0.3)	1,026 (0.3)	
	Transfer	3,376 (5.9)	18,681 (5.2)	
TST	Negative	4,745 (29.3)	14,461 (28.0)	<0.001
(67,942)	Positive	11,481 (70.7.2)	37,255 (72.0)	
AFB smear	Negative	23,936 (80.0)	82,602 (26.5)	<0.001
(341,807)	Positive	5,997 (20.0)	229,272 (73.5)	
Culture	Negative	6,775 (59.3)	22,103 (33.2)	<0.001
(78,004)	Positive	4,648 (40.7)	44,478 (66.8)	
Histopathology	Negative	884 (3.8)	2,184 (9.9)	<0.001
(44,855)	Positive	21,903(96.2)	19.884 (90.1)	
DOT	Not performed	28,727 (64.7)	153.811 (51.7)	<0.001
(341,831)	Performed	15,696 (35.3)	143,597 (48.3)	
Outcome status	Cure	30,186(71.5)	186,428 (70.1)	<0.001
(308,112)	Dropout	3,429 (8.1)	33,016 (12.4)	
	Death by TB	1,564 (3.7)	10,718 (4.0)	
	Death by other causes	3,024 (7.2)	11,046 (4.2)	
	Transfer	3,967 (9.4)	23,566 (8.9)	
	MDR-TB	28 (0.1)	1,140 (0.4)	

*Number of valid observations. **Pearson’s chi-squared test.

HIV/AIDS: human immunodeficiency virus/acquired immunodeficiency syndrome. TST: tuberculin skin test. AFB: acid-fast bacilli. DOT: Directly Observed Therapy. EPTB: Extrapulmonary tuberculosis. PTB: pulmonary tuberculosis.

### Comparative analysis: EPTB versus PTB

Although both PTB and EPTB were more prevalent in males, the proportion of females with EPTB was higher (p < 0.001). A significant difference was also observed in the age distribution between PTB and EPTB cases. Patients aged less than fourteen years old had EPTB more frequently than PTB (p < 0.001) (Table 1).

Among cases of EPTB, the proportion of white individuals and of individuals with more than seven years of education was significantly greater (p < 0.001 for both, Table 1). Alcoholism (p < 0.001), diabetes mellitus (p < 0.001) and mental illness (p < 0.001) occurred more frequently among patients with PTB, whereas the proportion of patients co-infected with HIV (p < 0.001) and having other co-morbidities diseases (p = 0.001) was greater among patients with EPTB (Table 2).

Table 2 demonstrates that the proportions of new cases, readmission cases following dropout, relapse cases, unknown admission type cases, and transfer cases were greater for EPTB than PTB. Although the percentages of positive and negative TST were similar between EPTB and PTB, the difference was statistically significant (p < 0.001; Table 2). PTB patients were less frequently subjected to tissue biopsy and histopathological examination for diagnosis (p < 0.001), but were more often treated under DOTS (p < 0.001). The number of deaths due to pulmonary forms of TB was greater than the number of deaths due to EPTB, and the number of deaths due to other causes was higher for EPTB (p < 0.001; Table 2).

Due to the significance found with the chi-squared test (Tables 1 and 2), all of the investigated variables were included in the hierarchical model and remained significant during the various stages of analysis as demonstrated in Tables 3 and 4.

**Table 3:** Hierarchical logistic regression of forms of TB and sociodemographic characteristics, Brazil, 2007 – 2011

**Level**	**Characteristics**		OR *	CI (95%) **
Level 1	Gender	Female	Reference	
		Male	0.77	0.76 – 0.78
	Age	0 – 14 years old	2.52	2.39 – 2.65
		15 – 24 years old	0.76	0.74 – 0.78
		25 – 34 years old	Reference	
		35 – 44 years old	1.11	1.08 – 1.14
		45 – 54 years old	0.99	0.96 – 1.02
		55 – 64 years old	1.00	0.96 – 1.03
		>60 years old	1.13	1.09 – 1.17
	Skin color/race	White	Reference	
		Black	0.75	0.72 – 0.77
		Brown	0.75	0.73 – 0.77
		Asian	0.82	0.75 – 0.90
		Indigenous	0.63	0.57 – 0.70
	Education	Illiterate	Reference	
		1 – 4 years	1.49	1.39 – 1.58
		5 – 8 years	2.00	1.88 – 2.13
		9 – 12 years	3.09	2.90 – 3.29
		>12 years	4.93	4.61 – 5.28
		Non-applicable	2.19	2.07 – 2.33
Level 2	Dwelling	Urban	Reference	
		Rural	0.82	0.79 – 0.85
		Peri-urban	0.89	0.78 – 1.01
	Institutionalization	No	Reference	
		Yes	0.61	0.59 – 0.64

*OR: Odds Ratio of EPTB outcomes compared to PTB **CI: confidence interval.

HIV/AIDS: human immunodeficiency virus/acquired immunodeficiency syndrome. EPTB: extrapulmonary tuberculosis. PTB: pulmonary tuberculosis.

**Table 4:** Hierarchical logistic regression of forms of TB and clinical characteristics, Brazil, 2007 – 2011

**Level**	**Characteristics**		OR *	CI (95%) **
Level 3	HIV/AIDS	Negative	Reference	
		Positive	2.15	2.09 – 2.21
	Alcoholism	No	Reference	
		Yes	0.45	0.43 – 0.46
	Diabetes	No	Reference	
		Yes	0.54	0.51 – 0.57
	Mental illness	No	Reference	
		Yes	0.88	0.82 – 0.95
	Other diseases	No	Reference	
		Yes	1.18	1.15 – 1.22
Level 4	Admission type	New cases	Reference	
		Relapse	0.65	0.62 – 0.69
		Readmission after dropout	0.39	0.37 – 0.42
		Unknown	0.66	0.53 – 0.81
		Transfer	1.31	1.24 – 1.37
	TST	Negative	Reference	
		Positive	1.08	1.02 – 1.13
	AFB smear	Negative	Reference	
		Positive	0.12	0.12 – 0.13
	Culture	Negative	Reference	
		Positive	0.64	0.60 – 0.67
	Histopathology	Negative	Reference	
		Positive	3.57	3.24 – 3.92
Level 5	DOT	Not performed	Reference	
		Performed	0.69	0.67 – 0.71
	Exit condition	Cure	Reference	
		Dropout	0.79	0.76 – 0.83
		Death by TB	0.73	0.68 – 0.79
		Death by other causes	1.05	1.00 – 1.12
		Transferal	1.24	1.18 – 1.30
		MDR-TB	0.43	0.27 – 0.68

*OR: Odds Ratio of EPTB outcomes compared to PTB **CI: confidence interval TST: tuberculin skin test. AFB: acid-fast bacilli. DOT: Directly Observed Therapy. EPTB: extrapulmonary tuberculosis. PTB: pulmonary tuberculosis.

Males and institutionalized patients were less likely to have EPTB (OR 0.77; 95% CI 0.76 – 0.78) and (OR 0.61; 95% CI 0.59 – 0.64), respectively (Table 3).

EPTB patients were more likely to be white, less than fourteen years old (OR 2.52; 95% CI 2.39 – 2.65), have more than 12 years of education (OR 4.93; 95% CI 4.61 – 5.28), and be HIV seropositive (OR 2.15; 95% CI 2.09 – 2.21) (Table 3).

Table 4 describes 3 levels of the hierarchical logistic regression model. Level 2 which corresponds to comorbidities show that alcoholism (OR 0.46; 95% CI 0.43 – 0.46), mental illness (OR 0.88; 95% CI 0.82 – 0.95), and diabetes mellitus (OR 0.54; 95% CI 0.51 – 0.57) occurred less frequently in patients with EPTB compared to those with PTB. The variables corresponding to level 4 associated with EPTB included admission due to transfer (OR 1.31; 95% CI 1.24 – 1.37), positive TST (OR 1.08; 95% CI 1.02 – 1.13), and positive histopathological examination (OR 3.57; 95% CI 3.24 – 3.92). The variables in level 4 that exhibited the least association with EPTB were positive AFB smear test result (OR 0.12; 95% CI 0.12 – 0.13) and culture performed (OR 0.64; 95% CI 0.60 – 0.37).

Regarding the outcome variables, treatment was administered under DOT less frequently in patients with EPTB than PTB (OR 0.69; 95% CI 0.67 – 0.71), and patients with EPTB were less likely to default during treatment than patients with PTB (OR 0.79; 95% CI 0.76 – 0.83) (Table 4).

## Discussion

In Brazil, although EPTB primarily affects adults, one-fourth of all cases of EPTB occurred in children less than 14 years of age. The age variable was not normally distributed in our dataset. Thus, we built different models including this variable as continuous and categorical. The model with the better fit was with categorical variable (Akaike Information Criterion (AIC) = 312853 to continuous and AIC = 310940 to categorical). Also, the choice of categories took into account those described in the “Revised international definitions in tuberculosis control” [20]. Other affected groups include whites, individuals with 5 to 8 years of formal education, residents of urban areas, and non-institutionalized individuals. Histopathological examination of affected sites and tissues is usually recommended for diagnosis, and a large number of samples from patients with EPTB are AFB positive. The low proportion of patients with extrapulmonary TB treated under DOT should alert the national TB control program to the possible underuse of DOT in patients with EPTB and potentially lower cure rates in such patients. The high rate of transfers suggests the difficulties or lack of knowledge associated with the management of patients with EPTB.

Limitations of our study include deficiencies in the secondary databases including, possible missing or incomplete data, and potential biases and errors during data entry. We analyzed data for patients with pulmonary and extrapulmonary forms of TB. Additional studies of patients with combined pulmonary and extrapulmonary TB are needed to understand the characteristics of such patients.

Nevertheless, because the number of TB cases in our study was large and national TB surveillance data in Brazil are reasonably complete, our statistical analysis is robust and remains significant. In addition, it is believed that EPTB might be underreported due to its difficult diagnosis, which requires a high degree of clinical suspicion and biopsy or culture for confirmation. Strengths of our study include its national scope, the use of a hierarchical logistic regression model, and its new contribution to the knowledge about EPTB in Brazil.

We found that the most frequent site of EPTB was the pleura, followed by the peripheral lymph nodes. In the US between 1993 and 2006, the lymph node was the most common site of extrapulmonary involvement, followed by the pleura [16]. Tuberculous pleurisy occurs frequently in association with PTB. Moreover, evidence suggests that pleural TB represents an early manifestation of primary infection by *Mycobacterium tuberculosis* (MTB) and may serve as a sentinel event in recent transmission studies [21].

The proportion of cases of EPTB relative to the other clinical forms of TB exhibits wide variation among different countries and seems to be higher in those with a low incidence of TB, such as the US (21%), Italy (32%), Japan (23%) and Australia (39%) [1]. The reasons for these differences are unknown but could be related to different epidemiological patterns and the proportions of persons with TB as a consequence of recent (as opposed to remote) infection [21]. It is believed that in these in developed countries the proportion of patients with PTB, primarily responsible for the transmission of TB to other persons is lower, under such conditions EPTB has become more perceptive in actual epidemiological studies. Also the subnotifications of EPTB due the intrinsic difficulties in EPTB diagnosis and the lack of access to adequate diagnostic infrastructure in high TB burden developing countries could have also contributed to the observed EPTB disparity between the TB high burden developing countries and low incidence developed countries.

Conversely, in countries with a high incidence of TB, EPTB usually represents a smaller fraction of all cases. In 2011, the proportion of EPTB was 19% in India, 4% in Indonesia, and 18% in Pakistan. Our study demonstrated that the proportion of EPTB was 12.6% which is similar to high TB burden countries [1,9].

The higher proportion of females in EPTB cases compared with PTB cases was statistically significant, confirming other studies [16,22-24]. The association between white race and EBTB found in the present study disagrees with the results found in a US study where 81% of cases of EPTB occurred among non-whites [16]. Investigators in the UK and Germany have reported that EPTB is more prevalent among particular racial groups, such as Asians, whereas it is less frequent among Caucasians [10,23]. In another Brazilian study of 606 patients with TB an association between EPTB and white race was also present [25]. The predominance of the white race also differs between the cases of PTB in Brazil, where individuals of brown and black ethnicity are most affected by PTB [10].

Our analysis also demonstrated that a higher education level was associated with a higher frequency of EPTB. In Brazil this result may be related to easier access to diagnostic and health services among more educated individuals, given that the diagnosis of EPTB often requires invasive sampling procedures with additional expense and risk, and the collection of such samples is more difficult in public health services of resource-constrained countries [26]. We were unable to identify other international studies examining educational level and extrapulmonary TB to compare our findings.

Regarding clinical factors, our analysis confirms many earlier reports [4,6,10,16,27] of the strong association between HIV co-infection and extrapulmonary TB. In a Brazilian study on TB-HIV coinfection between 2006 and 2010, EPTB occurred in 26.2% of HIV-infected patients [28]. In a recent meta-analysis, the pooled analysis showed a significant relationship between HIV infection and EPTB (OR:1.3, CI 1.05-1.6) [29].

The fact that 39.9% of the cases of EPTB were awaiting HIV test results or were not tested deserves particular attention. A smaller number of patients with TB who are tested for HIV correlates with a greater uncertainty relative to the prevalence of coinfection [30]. All patients who give consent must be tested for HIV, and mechanisms should be established to ensure that the results are systematically included in information systems as soon as they become available [30].

The most frequent type of admission of patients with EPTB to the health system was through transfers. This phenomenon may be related to the greater difficulty of health professionals in peripheral health units to confidently diagnose EPTB; thus, it is necessary to refer patients to other services for diagnostic confirmation or to begin treatment without confirmation based on a high degree of clinical suspicion and negative AFB smear results [31].

Mycobacterial cultures are not universally performed during evaluation of TB suspects in Brazil and are indicated only in specific instances, such as in cases of suspected EPTB or drug resistant TB and in vulnerable populations [32]. For this reason, cultures were performed in a larger proportion of cases of EPTB compared with PTB in our study, although cultures were positive in a smaller fraction of EPTB patients.

Anti-TB treatment was not conducted under DOT in 64.7% of the cases of EPTB, which is significantly higher compared with PTB cases. Among the recommendations for the treatment of EPTB, lack of adherence, poor absorption of anti-TB drugs, and drug resistance must be considered as possible reasons for the delayed or suboptimal response to appropriate treatment, and DOT is strongly recommended to enhance treatment completion and cure of the patient [33].

EPTB is an important health problem due to its high mortality and morbidity rates, both in developing and developed countries [34]. EPTB is less contagious, less frequent than PTB, and less well addressed by programs in developing countries, but improved diagnosis of extrapulmonary forms of TB and supervised treatment are essential prerequisites for optimizing care and better treatment outcomes [35].

Knowledge about determinants and characteristics of patients with EPTB from our study should motivate more investigation of extrapulmonary involvement to improve patient care and TB control in Brazil.

## Conclusions

As can be seen in our study, characteristics of EPTB often differ from those of PTB, as well as from those reported in countries with a low burden of TB. Therefore, studies aiming at these differences are important.

Beyond identification of EPTB cases, special diagnostic methods like aspirates and biopsies must be guaranteed but the resources to do them are limited in many parts of Brazil. Improved diagnostic capacity is essential for better case finding and management of EPTB. Because EPTB is less infectious to others in the community, it may receive less attention than smear-positive PTB from TB programs and health units with limited resources. More specific programs and resources are needed to improve the diagnosis, treatment and outcome of EPTB in Brazil.

## Competing interests

The author(s) declare that they have no competing interests.

## Authors’ contributions

All authors made substantive intellectual contributions to the study and read and approved the final manuscript. TG designed the study, analysed the data and drafted the manuscript. BR analysed the data and revised the manuscript. AB, JLJ and LWR critically reviewed and revised the manuscript. ELM was involved in the acquisition of the data, analysis and interpretation of data, and revision of the manuscript. All authors read and approved the final manuscript.

## Pre-publication history

The pre-publication history for this paper can be accessed here:

http://www.biomedcentral.com/1471-2334/14/9/prepub
